# Data from molecular dynamics simulations in support of the role of human CES1 in the hydrolysis of Amplex Red

**DOI:** 10.1016/j.dib.2016.01.031

**Published:** 2016-01-28

**Authors:** Giulio Vistoli, Achim Treumann, Thomas von Zglinicki, Satomi Miwa

**Affiliations:** a Department Pharmaceutical Sciences, University of Milan, via Mangiagalli, 25, I-20133 Milan, Italy; b Newcastle University Protein and Proteome Analysis, Devonshire Building, Devonshire Terrace, Newcastle upon Tyne NE1 7RU, UK; c Institute for Cell & Molecular Biosciences and Newcastle University Institute for Ageing, Ageing Research Laboratories, Campus for Ageing and Vitality, Newcastle University, Newcastle upon Tyne NE4 5PL, UK

## Abstract

This data article contains the results of molecular dynamics (MD) simulations performed to assess the stability of the previously computed complex between the hCES1 structure and the Amplex Red (AR) substrate (Miwa et al., 2015) [1] and to compare the dynamic behavior of this complex with that of the corresponding hCES1-deacetylAR product. The study involves both standard molecular dynamics (MD) and steered (SMD) simulations to offer a quantitative comparison of the stability for the two complexes. With regard the standard MD runs, the data article graphically reports the r.m.s.d. profile of the ligand׳s atoms as well as the dynamic behavior of key contacts involving the catalytic Ser221 residue. The SMD simulations provide a comparison of the pull forces required to undock the two ligands and reveal that Van der Waals and hydrophobic interactions play a key role in complex stabilization.

## Specifications Table

**Table:** 

Subject area	*Biology*
More specific subject area	*Molecular modeling studies of protein-ligand complexes*
Type of data	*Data extracted from MD trajectories and represented by plots within the text*
How data was acquired	*Molecular Dynamics (MD) and Steered MD (SMD) simulations using NAMD*
Data format	*Binding features and structural parameters graphically analyzed*
Experimental factors	*Starting complexes computed by docking simulations*
Experimental features	*5 ns standard all-atoms MD runs plus 1 ns Steered MD simulations*
Data source location	*Milan, Italy*
Data accessibility	*Data are within this article*

## Value of data

• The MD runs provided here confirmed the stability of the computed CES1-AR complex.
• The SMD runs evidenced notable differences between the undocking processes of the substrate and product.
• The SMD runs emphasized the key role played by Van der Waals and hydrophobic interactions during the undocking pathways.
• Presented data confirmed that suitably targeted MD simulations can be useful to predict the dynamic behavior of enzyme-substrate complexes.
• The combination of MD and SMD runs allow the complex stability to be assessed also investigating the specific role of each interaction type.

## 1. Data

The data were generated by two sets of all-atoms MD simulations involving the hCES1 structure in complex with both Amplex Red (AR) and the corresponding enzymatic product deacetylAR. The first set involved standard 5 ns MD runs with a view (a) to assess the stability of the hCES1-AR complex and (b) to reveal the egress process for the enzymatic product (at least in its initial phase). The second set involved steered MD runs (SMD) in order to offer a quantitative comparison of the stability of the two simulated complexes and to reveal the energy factors governing the undocking processes.

**Fig. 1:**
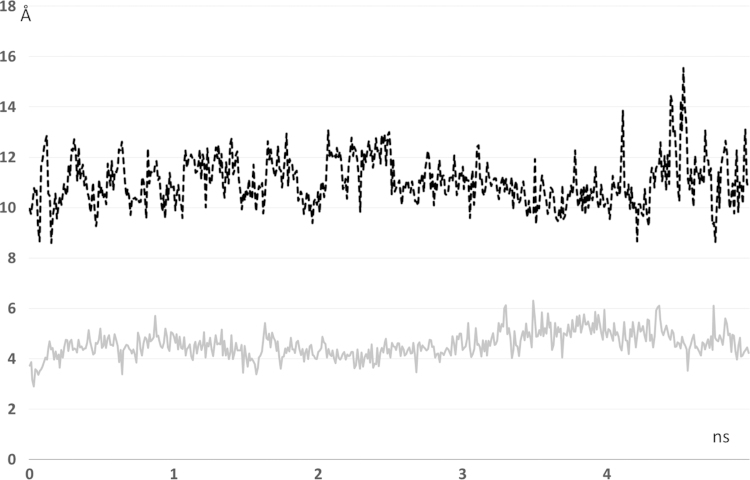
Dynamic profiles of the distances between the catalytic Ser221 residue and the labile amide function for the hCES1-AR complex (gray line) and between Ser221 and the remaining amine function for the hCES1-deacetylAR complex (dashed black line).

**Fig. 2:**
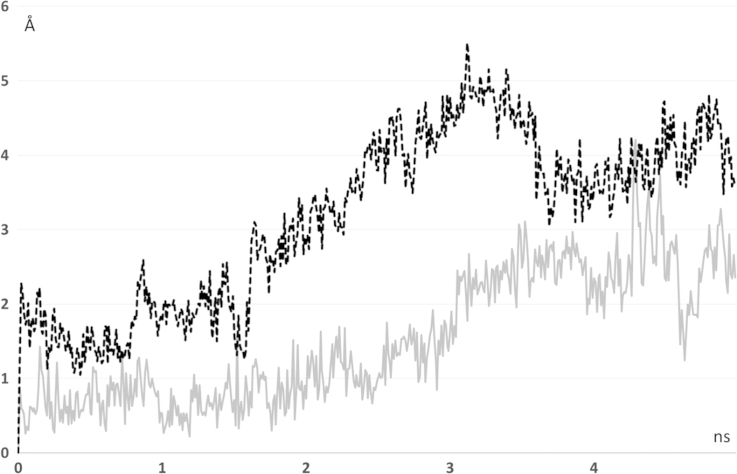
Dynamic profiles of the r.m.s.d. values as computed for the ligand atoms only (hCES1-AR complex=gray line and hCES1-deacetylAR complex=dashed black line).

**Fig. 3:**
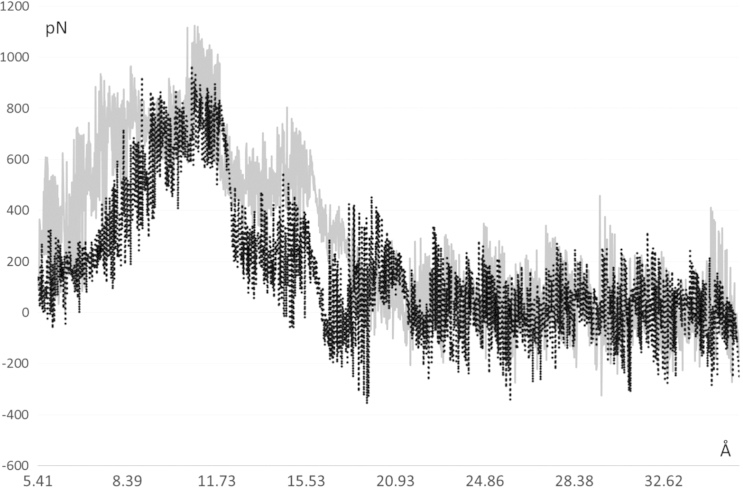
Pull force profiles along the *x* axis for the ligand undocking (hCES1-AR complex=gray line and hCES1-deacetylAR complex=dashed black line).

**Fig. 4:**
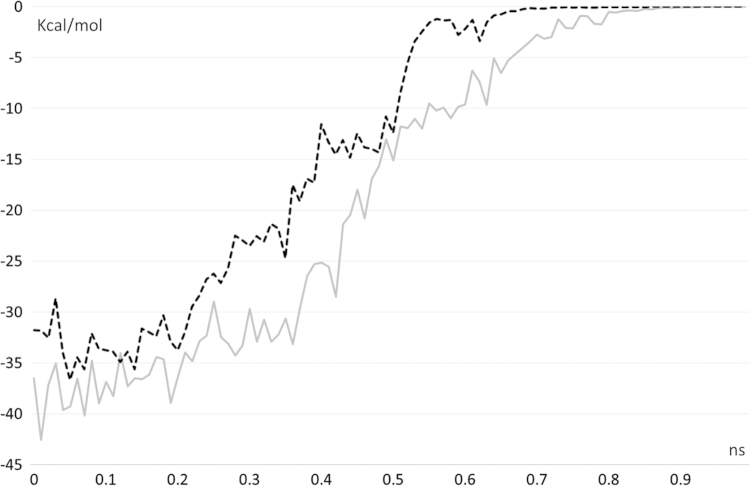
Energy profile for the Van der Waals interactions as computed by SMD runs using the CHARMM L–J term (hCES1-AR complex=gray line and hCES1-deacetylAR complex=dashed black line).

**Fig. 5:**
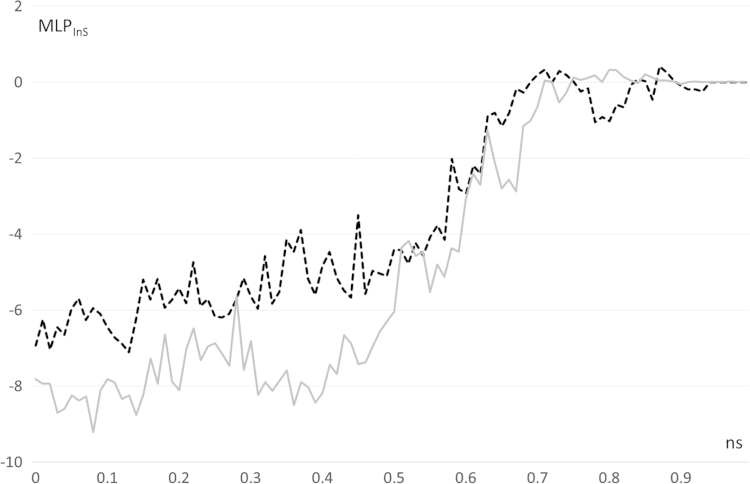
Energy profile for the hydrophobic interactions as computed by SMD runs using the MLP_InS_ interaction score (hCES1-AR complex=gray line and hCES1-deacetylAR complex=dashed black line, MLP_InS_ is dimensionless).

Fig. 1, Fig. 2 compares the different dynamic of substrate and product as revealed by their distance profiles with the catalytic Ser221 (see Fig. 1) and the r.m.s.d. values as computed considering the ligand atoms only. Fig. 3 compares the pull force profiles along the *x* axis of the undocking processes for the two simulated complexes as derived by SMD simulations. The analysis of the interaction scores as computed during the SMD runs reveals that the undocking processes are mainly governed by Van der Waals and hydrophobic interactions as parameterized by CHARMM Lennard Jones (L–J) energies (see Fig. 4, [2]) and by MLP Interaction scores (MLP_InS_ see Fig. 5, [3]), while ionic interactions were found to be negligible and roughly constant during the SMD runs (data not shown).

## 2. Experimental design, materials and methods

### 2.1 Complex preparation

The optimized hCES1-AR complex was computed by docking simulations using PLANTS as described in the reference paper [1]. The hCES1-deacetylAR complex was prepared by manually transforming the hCES1-AR complex and was minimized by keeping fixed all atoms outside a 10 Å radius sphere around the bound ligand. Due to their net positive charge equal to +5, the two optimized complexes were neutralized by adding 5 chlorine ions using the SODIUM tool [4] as implemented in the VEGA suite of programs [5]. The neutralized complexes were then inserted into a 80 Å side cubic box of water molecules so as to generate hydrated complexes containing about 13,500 solvent molecules. The so obtained systems were finally minimized to optimize the position of solvents and ions and underwent the following MD and SMD simulations.

### 2.2 MD simulations

The two prepared complexes underwent 5 ns canonical all-atoms MD simulations with the following key features: (a) the simulation space was stabilized by applying periodic boundary conditions (90 Å×90 Å×90 Å); (b) the integration of Newton׳s equation was performed by using r-RESPA method (every 4 fs for long-range electrostatic forces, 2 fs for short-range nonbonded forces, and 1 fs for bonded forces); (c) the Particle Mesh Ewald (PME) summation method (80×80×80 grid points) was utilized to calculate the long-range electrostatic potential; (d) the temperature was maintained at 300±10 K by applying the Langevin׳s algorithm; (e) Lennard–Jones (L–J) interactions were calculated with a cut-off of 10 Å and the pair list was updated every 20 iterations; (e) a frame was memorized every 10 ps, thus producing 500 frames; and (f) no constraints were imposed to the systems. The simulations were carried out in two phases: an initial period of heating from 0 K to 300 K over 300,000 iterations (300 ps, i.e. 1 K/ps) and the production phase of 5 ns.

### 2.3 SMD simulations

The same optimized complexes underwent 1 ns steered MD simulations during which the bound ligands were pulled in the *x* axis along with the egress direction of the catalytic pocket as already determined by previous MD studies. The SMD runs had the same general characteristics already described for the canonical MD simulations, while the spring constant was equal to 5 kcal/mol/Å^2^ with a pulling velocity of 0.003 nm/ps. The mentioned minimizations were performed using the conjugate gradient algorithm until the r.m.s. gradient was smaller than 0.01 kcal mol^−1^ Å^−1^. All calculations were carried out by using Namd2.10 [6] with the force-field CHARMm v22 [2], [4] and the Gasteiger׳s atomic charges.
